# Clinico-epidemiological profile and quality of life of PLWHA in Central India

**DOI:** 10.1186/1471-2334-12-S1-P79

**Published:** 2012-05-04

**Authors:** Namita N Deshmukh, Jyotsna S Deshmukh, Sanjay S Kubde, Sushama S Thakre, Mohan B Khamgaonkar, Suresh Ughade

**Affiliations:** 1 Indira Gandhi Govt. Medical College, Nagpur, India; 2 Government Meical College, Nagpur, India

## Background

Advances in drug therapy have dramatically extended the life of people living with HIV/AIDS (PLWHA). Empirical evidence shows that as the HIV disease progresses, quality of life deteriorates. PLWHA face physiological, physical, psychological and socio-cultural problems that are caused by these factors affect quality of life. The purpose of this study was to study the clinico-epidemiological profile and assess quality of life of PLWHA.

## Methods

Study involved cross sectional assessment of 754 PLWHA above 18 years of age and having treatment duration ≥ 6 months. Socio-demographic characteristics and clinical profile which included associated morbidities, WHO clinical staging and CD4 counts of the patients were studied. Quality of life was assessed with WHO QOL BREF instrument. Chisquare test, Z-test, Pearson’s correlation coefficient, ANOVA with Bonferroni post-hoc test and Multiple Logistic Regression were used for analysis.

## Results

Out of 754 study subjects, 61.01% were males, 67.24% were married and 20.56% were widowed, 71.75% patients belonged to lower class, 58.22% patients were symptomatic and 22.2% patients suffered from peripheral neuropathy. Scores in the independence domain were highest and those in the physical domain were the lowest. Inter-domain consistency was found to be good (p=0.001). Age ≥ 50 years (OR 5.71, p=0.001), female sex (OR 2.1, p=0.010), illiteracy (OR 1.98, p=0.045), no spouse support (OR 3.06, p=0.001) and higher WHO clinical staging (OR 1.3, p=0.042) were found to be the major risk factors for poor quality of life.

## Conclusions

Though anti-retroviral therapy has prolonged life of PLWHA, their quality of life remains poor.

